# A Plant’s Electrical Parameters Indicate Its Physiological State: A Study of Intracellular Water Metabolism

**DOI:** 10.3390/plants9101256

**Published:** 2020-09-23

**Authors:** Cheng Zhang, Yanyou Wu, Yue Su, Deke Xing, Yi Dai, Yansheng Wu, Lei Fang

**Affiliations:** 1 Key Laboratory of Modern Agricultural Equipment and Technology, Ministry of Education, College of Agricultural Engineering, Jiangsu University, Zhenjiang 212013, China; 2111816013@stmail.ujs.edu.cn (C.Z.); xingdeke@ujs.edu.cn (D.X.); 2 State Key Laboratory of Environmental Geochemistry, Institute of Geochemistry, Chinese Academy of Sciences, Guiyang 550081, China; wuyansheng@mail.gyig.ac.cn (Y.W.); fanglei@mail.gyig.ac.cn (L.F.); 3 Department of Agricultural Engineering, Guizhou Vocational College of Agriculture, Qingzhen 551400, China; suyue09136@163.com (Y.S.); daiyi96@126.com (Y.D.)

**Keywords:** electrical information, bioenergetics, plant physiological state, monitor, intracellular water

## Abstract

Almost all of a plant’s life activities involve electrochemical reactions. Plant electrical parameters respond quickly to environmental changes and are closely related to physiological activities. In this study, the theoretical intrinsic relationships between clamping force and leaf impedance (Z) or capacitive reactance (Xc) and capacitance (C) were revealed as 3-parameter exponential decay and linear models based on bioenergetics, respectively, for the first time. Leaf electrical characteristics including intrinsic impedance (IZ), capacitive reactance (IXc), capacitance (IC) and specific effective thickness (d) were successfully detected using the above-mentioned relationships and were used to manifest plant metabolic activity. The intracellular water-holding capacity (IWHC), water-use efficiency (IWUE), water-holding time (IWHT) and water transfer rate (WTR) of plant leaves were defined on the basis of IZ, IXc, IC and d, and applied to reflect the intracellular water metabolism. The results demonstrated that the leaves of *Broussonetia papyrifera* plants grown in agricultural soil had higher IC, d, IWHC, WTR, water content values and lower IZ, IXc values than those grown in moderately rocky desertified soil. The leaf IC, d, IWHC, WTR and water content values of herbaceous plants were higher than those of woody plants. *Solanum tuberosum* L. had higher leaf IC, d, IWHC and WTR values, but exhibited lower IZ, IXc, IWUE and IWHT values than *Capsicum annuum* L. This study highlighted that a plant’s electrical parameters based on bioenergetics clearly indicate its physiological process—e.g., the intracellular water metabolism.

## 1. Introduction

Almost all life activities in plants involve charge separation, electron movement, and proton and dielectric transport [1]. The electrical properties of plant cells are derived from the cell membrane which forms a double electric layer. The main components of the cell membrane, membrane lipids, which can be regarded as an insulating layer, have high electrical resistivity, enabling the plant cell to store electric charge [1,2]. The electrical parameters of plants are closely related to the plants’ life activities, including their metabolism of substances and energy, development, stress resistance and signal transduction. When plant cells are damaged by abiotic or biotic stress, changes in structure, composition and ion permeability are evoked immediately, resulting in significant changes in their electrical parameters [2,3,4,5,6,7]. Changes in a plant’s electrical parameters are considered to be the fastest plant responses to environmental changes [8]. Most plant electrical parameters response to various stimuli and show low reproducibility [9,10]. Currently, the insertion of two electrodes into the stem or leaf is the traditional approach used for measuring electrical parameters in plants [11,12]. However, this method is unstable, difficult to manipulate and has low reliability. In addition, the plant electrical parameters lack representativeness, reproducibility and comparability, and can easily be directly influenced by needling injuries, environment changes, observer operations and other factors. Therefore, there is an urgent need to develop a method for monitoring the intrinsic electrical parameters in plants with high reproducibility.

Abiotic or biotic stimuli, including drought, salt, cold, diseases and insect pests, can rapidly trigger plant electrical activities [4,6,8,10]. Could the intrinsic relationships between environmental stimuli and electrical parameters be used to assess the intrinsic electrical parameters in plants or to evaluate their life phenomena? Can these intrinsic relationships be described by corresponding physical mechanism models? The impedance and capacitance values of plants have always been used to evaluate the water status, maturity and growth of plants [13,14,15,16,17,18]. Moreover, mesophyll cells have long been regarded as concentric sphere capacitor with both inductor and resistor functions, due to their specific composition and structure [19,20]. The ions, ion groups and electric dipoles in mesophyll cells are electrolytes, which are most strongly related to electrical processes [21,22]. Interestingly, Guo et al. [23] reported that the capacitance (C) values of maize leaves increased with clamping force. The significant variation in this electrical parameter was attributed to changes of the electrolyte concentration in mesophyll cells stimulated by variable clamping forces. However, this intrinsic relationship between the clamping force and the C of plant leaves has yet to be precisely revealed. This intrinsic mechanism or relationship between clamping forces and the C of plant leaves has not yet been revealed. Thus, it is of great practical significance to reveal the intrinsic mechanism linking clamping force and electrical parameters to provide a rapid, accurate and real-time method for monitoring the physiological state of plant leaves.

Most (95–99%) of the water in plant leaves dissipates through transpiration, but a small amount (1–5%) is retained in leaf cells to support plant growth [1]. The metabolic utilization of this precious intracellular water (1–5%) is of paramount importance to many physiological and biochemical processes, including photosynthesis, respiration, organic synthesis and decomposition [24,25]. Many techniques that are capable of monitoring leaf water content and water-use efficiency, including spectrometry, stable oxygen and hydrogen isotopes, water potential measurements and drying [26,27]. However, the leaf water content or water-use efficiency does not directly reflect the dynamic status of intracellular water in plant leaves, and the limitations of such methods in terms of their field use, cost, complexity and accuracy have limited their application in situ. To the best of our knowledge, no work to directly and quantitatively detect the intracellular water status of plant leaves has been conducted to date. Fully expanded leaves, which account for a high proportion of plant biomass, reflect plant water metabolism. Importantly, the water metabolism in plant leaves affects the concentration of electrolytes (ions, ion groups and electric dipoles) in leaf cells, and is thus accompanied by vigorous electrical activity. The leaf impedance (Z), capacitive reactance (Xc) and capacitance (C) are related to the concentration of ions, ion groups and electric dipoles, and intracellular water metabolism causes variations in the electrolyte concentration.

In this study, the intrinsic mechanisms and physical models relating clamping force and the Z, Xc, and C values of plant leaves were revealed for the first time. The leaf Z and C values of various plants were measured under different clamping forces using an LCR tester, which is a device for determining inductance (L), capacitance (C) and impedance (R). Moreover, the leaf Xc values were calculated from the leaf C. Subsequently, the intrinsic impedance (IZ), capacitive reactance (IXc), capacitance (IC) and specific effective thickness (d) of the intrinsic electrical parameters in the plant leaves were successfully obtained using the respective mechanism equations. The intracellular water-holding capacity (IWHC), water-use efficiency (IWUE), water-holding time (IWHT) and water-transfer rate (WTR), all key intracellular water metabolism indices in plant leaves, were defined based on the intrinsic electrical parameters, and applied to reflect the intracellular water metabolism. This study aimed to reveal the intrinsic mechanical relationships between exogenous stimuli and leaf Z, Xc, and C, as well as to provide a novel technique for rapidly monitoring the physiological states of plants.

## 2. Theories

### 2.1. Intrinsic Mechanism Relationships of Clamping Force (F) and Leaf Z, Xc, and C

**Figure 1:**
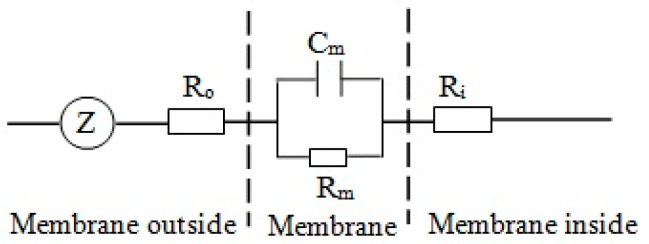
Simplified equivalent circuit of a plant cell. Z: impedance, C_m_: capacitance of the membrane, R_m_: resistance of the membrane, R_o_: resistance of the membrane outside, R_i_: resistance of the membrane inside.

Almost all life activities in plants are closely related to electrical processes [1]. The cell membrane of a mesophyll cell has strictly selective permeability to various ions, ion groups and electric dipoles, and the electrolyte solutions on the two sides of the cell membrane form a specific conductive state. The inside and outside of the cell membrane can be simulated as a capacitor, where the electrolyte solution on both sides of the membrane is equivalent to the two plates of the capacitor, and the cell membrane is equivalent to the intermediate medium of the capacitor. Moreover, organelles, such as vacuoles and cytoplasm in cells, are equivalent to resistors. Thus, a mesophyll cell can be modeled as a concentric sphere capacitor with both inductor and resistor functions [8,9,21]. The simplified equivalent circuit of a mesophyll cell is illustrated in Figure 1.

In this study, the ions, ion groups, and electrical dipoles in a plant leaf were used as electrolytes, and a parallel-plate capacitor sensor was formed by placing the leaf between the two plates of the parallel-plate capacitor. The leaf Z, Xc, and C values varied with the electrolyte concentration in the leaf, and the variation in the electrolyte concentration was caused by the water status of the leaf cells [17]. Different clamping forces, which can be considered different exogenous stimuli, inevitably changed cell membrane permeability, causing the ion, ion group, and electric dipole concentrations of the leaf cells to change instantly. Different clamping forces were achieved by adding iron blocks to the experimental setup.

The concentrations of electrolytes responding to Z inside and outside the cell membrane determine the plant leaf Z. External stimuli change the membrane permeability of electrolytes and affect their concentrations inside and outside the cell membrane. Under different clamping forces, the membrane permeability of electrolytes responding to Z in the plant cell membrane changes. According to bioenergetics, the Nernst equation can be used to quantitatively describe the potential of ions, ion groups and electric dipoles inside and outside the cell membrane [21,22]. Thus, the concentration differences in electrolytes responding to Z inside and outside the cell membrane obey the Nernst equation and can be expressed as follows:

(1) $$E-E^{0}=\frac{R_{0}T}{n_{Z}F_{0}\;}\ln\frac{Q_{i}}{Q_{o}}$$

where E: the electromotive force (V), E^0^: the standard electromotive force (V), R_0_: the gas constant (8.314570 J K^−1^ mol^−1^), T: the thermodynamic temperature (K), Q_i_: the concentration of electrolytes responding to Z inside the cell membrane (mol L^−1^), Q_o_: the concentration of electrolytes responding to Z outside the cell membrane (mol L^−1^), F_0_: Faraday constant (96,485 C mol^−1^),and nz: the number of transferred electrolytes (mol).

The internal energy of the electromotive force can be converted into pressure work, with a direct relationship, PV = a E:

(2) $$\mathrm{PV}=\mathrm{aE}={a\;E}^{0}+\frac{a\;R_{0}T}{n_{Z}F_{0}\;}\ln\frac{Q_{i}}{Q_{o}},$$

where P: the pressure intensity on the leaf cells (Pa), a: the energy conversion coefficient of the electromotive force and V = the cell volume (m^3^). Further, $P=\frac{F}{S}$, where F: the clamping force (N) and S: the effective area of the electrode plate (m^2^). F can be calculated by the gravity formula:

(3) F=(M+m)g,

where M: the iron block mass (kg), m: the mass of the plastic rod and the plate electrode (kg) and g: 9.8 N/kg.

For mesophyll cells, the sum of Q_o_ and Q_i_ is certain. Q_i_ is directly proportional to the conductivity of electrolytes that respond to Z, and the conductivity is the reciprocal of Z. Hence, $\frac{Q_{i}}{Q_{o}}$ can be expressed as $\frac{Q_{i}}{Q_{o}}=\frac{\frac{J_{0}}{Z}}{Q-\frac{J_{0}}{Z}}=\frac{J_{0}}{Q\;Z-J_{0}}$, where J_0_: the ratio coefficient of the conversion between Q_i_ and Z and Q is Q_o_ + Q_i_. Therefore, Formula (2) can be transformed into Formula (4):

(4) $$\frac{V}{S}F={a\;E}^{0}-\frac{{a\;R}_{0}T}{n_{Z}F_{0\;}}\ln\frac{\mathrm{QZ}-J_{0}}{J_{0}},$$

Formula (4) can be rewritten as:

(5) $$\frac{{a\;R}_{0}T}{n_{Z}F_{0\;}}\ln\frac{\mathrm{QZ}-J_{0}}{J_{0}}={a\;E}^{0}-\frac{V}{S}F,$$

and

(6) $$\ln\frac{\mathrm{QZ}-J_{0}}{J_{0}}=\frac{n_{Z}F_{0}E^{0}}{\mathrm{RT}}-\frac{{\mathrm{Vn}}_{Z}F_{0}}{S\;a\;\mathrm{RT}}F,$$

Formula (6) takes the exponents of both sides:

(7) $$\frac{\mathrm{QZ}-J_{0}}{J_{0}}=e^{\frac{n_{Z}F_{0}E^{0}}{R_{0}T}}e^{\left(-\frac{{\mathrm{Vn}}_{Z}F_{0}}{{\mathrm{Sa}\;R}_{0}T}F\right)},$$

Further,

(8) $$\;Z=\frac{J_{0}}{Q}+\frac{J_{0}}{Q}e^{\frac{n_{Z}F_{0}E^{0}}{R_{0}T}}e^{\left(-\frac{{\mathrm{Vn}}_{Z}F_{0}}{{\mathrm{Sa}\;R}_{0}T}F\right)},$$

As d$=\frac{V}{S}$, Formula (8) is transformed into:

(9) $$\;Z=\frac{J_{0}}{Q}+\frac{J_{0}}{Q}e^{\frac{n_{Z}F_{0}E^{0}}{R_{0}T}}e^{\left(-\frac{{d\;n}_{Z}F_{0}}{{a\;R}_{0}T}F\right)},$$

For the same leaf tested in the same environment, the d, a, E^0^, R_0_, T, nz, F_0_, Q and J_0_ of Formula (8) are constants. Let $y_{0}$=$\frac{J_{0}}{Q}$, k_1_=$\frac{J_{0}}{Q}e^{\frac{n_{Z}F_{0}E^{0}}{R_{0}T}}$, and b_1_=$\frac{{d\;n}_{Z}F_{0}}{{\;a\;R}_{0}T}$; then, the intrinsic mechanical relationships of leaf Z and F are

(10) $$Z=y_{0}+k_{1}\;e^{-b_{1}F},$$

where y_0_, k_1_ and b_1_ are the model parameters.

When F = 0, the intrinsic impedance (IZ) of the plant leaves can be obtained:

(11) $$\mathrm{IZ}=y_{0}+k_{1},$$

With the same Z, the intrinsic mechanical relationships of leaf Xc and F are revealed (Additional File 1):

(12) $$\mathrm{Xc}=p_{0}+k_{2}{\;e}^{-b_{2}F},$$

where p_0_, k_2_, and b_2_ are the model parameters.

When F = 0, the intrinsic capacitive reactance (IXc) of the plant leaves can be calculated as:

(13) $$\mathrm{IXc}=p_{0}+k_{2},$$

Subsequently, the intrinsic capacitance (IC) of the plant leaves can also be obtained:

(14) $$\mathrm{IC}=\frac{1}{2\pi f\mathrm{IXc}},$$

where π: 3.1416, f: the frequency and IXc: the intrinsic capacitive reactance.

According to the first law of thermodynamics, the work done by the clamping force obeys the Gibbs free energy equation:

(15) ΔG=ΔH+PV,

where ΔG: Gibbs free energy (J), ΔH: the internal energy of the leaf cell system (J), P: the pressure intensity of the leaf cells (Pa) and V: the cell volume (m^3^). P can be calculated by the pressure intensity formula:

(16) $$P=\frac{F}{S},$$

where F: the clamping force (N) and S: the effective area of the electrode plate (m^2^).

Mesophyll cells can be regarded as concentric sphere capacitors for which the capacitor energy is

(17) $$W=\frac{1}{2}U^{2}C,$$

where W: the capacitor energy (J), U: the test voltage (V), and C: the physiological capacitance (pF).

According to energy conservation theory, a capacitor’s energy is equal to the work converted by the Gibbs free energy, i.e., W = ΔG. The leaf C and clamping force (F) relationship model can thus be obtained:

(18) $$C=\frac{2\Delta H}{U^{2}}+\frac{2V}{{\mathrm{SU}}^{2}}F,$$

It is assumed that d represents the specific effective thickness of the plant leaves; therefore, d$\;=\frac{V}{S}$. Formula (18) is transformed into Formula (19):

(19) $$C=\frac{2\Delta H}{U^{2}}+\frac{2d}{U^{2}}F,$$

Let $x_{0}=\frac{2\Delta H}{U^{2}}$ and $h=\frac{2d}{U^{2}}$; Formula (19) is then transformed into Formula (20):

(20) $$C=x_{0}+\mathrm{hF},$$

Formula (20) is a linear model, where x_0_ and h are the model parameters.

As $h=\frac{2d}{U^{2}}$, the specific effective thickness (d) of the plant leaves can be calculated as:

(21) $$d\;=\frac{U^{2}h}{2},$$

### 2.2. Intracellular Water Utilization Parameters

The cell is a spherical structure, and its growth is closely related to an increase in volume. The C of plant leaf cells can be calculated using a formula for concentric spherical capacitors:

(22) $$\mathrm{Cc}=\frac{4\pi \varepsilon \;R_{1}R_{2}}{R_{2}-R_{1}},$$

where π: 3.1416, Cc: the capacitance of the concentric spherical capacitor (pF), ε: the dielectric constant of electrolytes, *R_1_*: the outer sphere radius (m) and *R_2_*: the inner sphere radius (m). For a plant cell, *R_2_* − *R_1_* is the thickness of the cell membrane. *R_1_* ≈ *R_2_*, ε and the thickness of the cell membrane are constant. Therefore, the cell volume (Vc) has the following relationship with the cell’s C:

(23) $$\mathrm{Vc}\;=\;\alpha \sqrt{C^{3},}$$

The cell volume is positively correlated with the volume of the vacuole, and the main component of the vacuole and cytoplasm is water. In other words, the water-holding capacity of cells is directly proportional to $\sqrt{C^{3}}$. Therefore, $\sqrt{C^{3}}$ can represent the water-holding capacity of plant leaves. The intracellular water-holding capacity (IWHC) of plant leaves can be obtained according to Formula (24):

(24) $$\mathrm{IWHC}\;=\sqrt{{\left(\mathrm{IC}\right)}^{3}},$$

The specific effective thickness (d) of plant leaves represents cell growth, and the water-holding capacity supports plant cell growth. Therefore, the intracellular water-use efficiency (IWUE) of leaves is represented by Formula (25):

(25) $$\mathrm{IWUE}\;=\frac{d}{\mathrm{IWHC}},$$

According to Ohm’s law, I_Z_: U/Z, where I_Z_: the physiological current (A), U: the test voltage (V), and Z: the physiological impedance (Ω). At the same time, the current is equal to the product of the capacitance and the differential of voltage in time, as shown in Formula (26):

(26) $$I_{Z}\;=\;\mathrm{IC}\times \int \mathrm{dU},$$

After the integral transformation, the current time is the product of the capacitance and impedance. Therefore, the intracellular water-holding time (IWHT) of plant leaves is represented by Formula (27):

(27) IWHT=IC×IZ,

Furthermore, the dynamic water transfer rate (WTR) of plant leaves is calculated by Formula (28):

(28) $$\mathrm{WTR}=\frac{\mathrm{IWHC}}{\mathrm{IWHT}},$$

## 3. Results

### 3.1. Intracellular Water Utilization of B. Papyrifera Grown in Two Habitats

**Figure 2:**
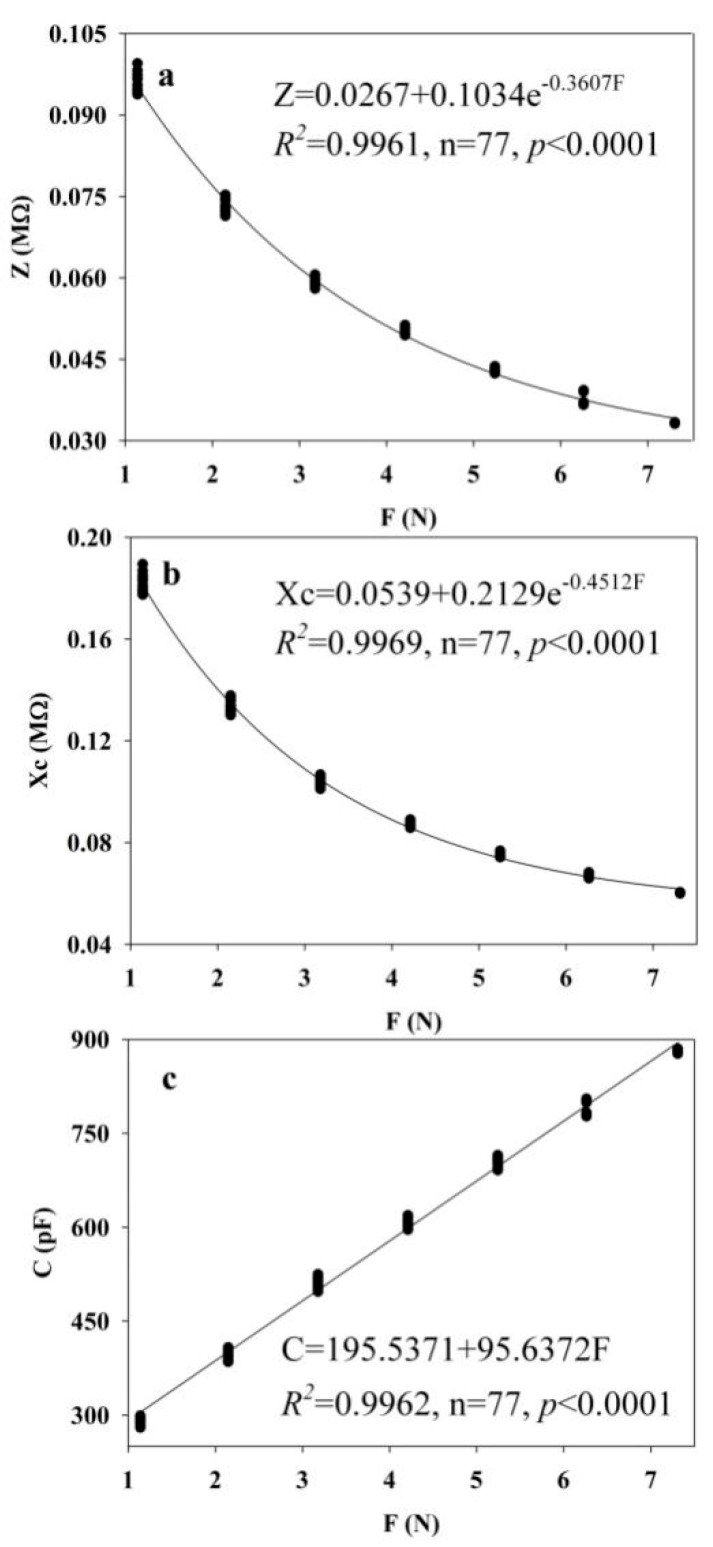
Fitting equations of the relationships between clamping force (F) and leaf Z (**a**), Xc (**b**) and C (**c**) of the fifth expanded leaf of the first branch of *B. papyrifera* grown in agricultural soil.

Figure 2 randomly lists the fitting curves and equations for the relationships between the Z (Figure 2a), Xc (Figure 2b), and C (Figure 2c) values of a *Broussonetia papyrifera* leaf grown in agricultural soil with the clamping force. The results showed that the leaf Z, Xc, and C values correlated well with the clamping force. Subsequently, the fitting equation coefficients for both the clamping force and the leaf Z, Xc and C in *B. papyrifera* plants grown in agricultural and moderately rocky desertified soils were calculated separately (Additional File 2). Moreover, all the *p* values were lower than 0.0001. This result further showed that the fitting equations of Z–F, Xc–F and C–F had good correlations, which highlights the existence of the intrinsic mechanical relationships of F and leaf Z, Xc and C. These fitting equations were also verified using *Brassica napus* samples in a phytotron, where the correlation coefficients (*R*^2^) were all over 0.9900 (Additional File 3). The water-use parameters for the two *B. papyrifera* growth conditions were obtained using the corresponding parameters of the fitting equations (Table 1).

**Figure 3:**
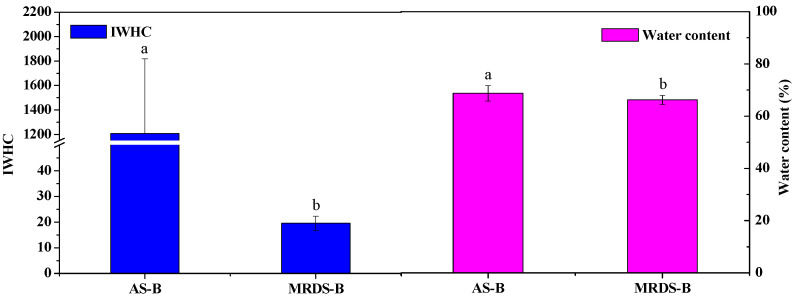
IWHC and water content of *B. papyrifera* in the two habitats. Values indicate the mean ± SD, n = 12. Lower case letters indicate significant differences at a 5% level (*p* < 0.05). AS-B: *B. papyrifera* grown in agricultural soil, MRDS-B: *B. papyrifera* grown in moderately rocky desertified soil.

As shown in Table 1 and Figure 3, the leaf IZ, IXc, IC, d, IWHC, IWHT, WTR and water content values of *B. papyrifera* plants grown in the two habitats were significantly different (*p* < 0.05). The leaf IC, d, IWHC, WTR and water content values of the *B. papyrifera* plants grown in the agricultural soil were significantly (*p* < 0.05) higher than those of the *B. papyrifera* plants grown in moderately rocky desertified soil, and had lower (*p* < 0.05) IZ and IXc values. The results showed that the water status of the leaves of *B. papyrifera* plants grown in the better soil was good; the plant become vigorous, and the leaf IC, d, IWHC and WTR values were higher. Due to the poor water status of the leaves of *B. papyrifera* plants grown in moderately rocky desertified soil, the water supply time (IWHT) of the leaf organs was maintained by decreasing the WTR, resulting in a high IWHT. However, the leaf IWUEs of *B. papyrifera* in the two habitats were not significantly different, which demonstrated that there was little difference in the intracellular water-use efficiency of leaves in the same plant.

### 3.2. Intracellular Water Utilization of Herbaceous and Woody Plants

**Figure 4:**
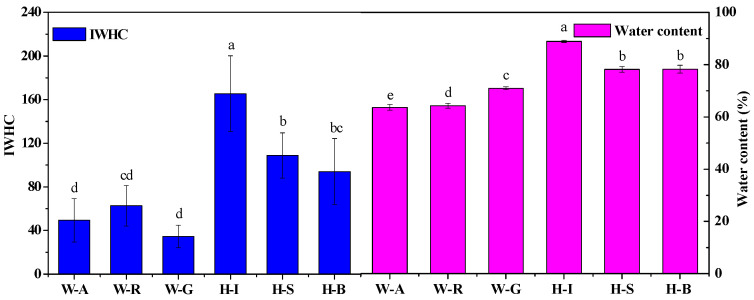
Intracellular water-holding capacity (IWHC) and water content of the six plants. Values indicate the mean ± SD, n = 12. Lower case letters indicate significant differences at a 5% level (*p* < 0.05). W-A: Woody plant-*Amygdalus persica*, W-R: Woody plant-*Rhus chinensis*, W-G: Woody plant- *Ginkgo biloba*, H-I: Herbaceous plants-*Ipomoea batatas*, H-S: Herbaceous plants-*Senecio scandens*, H-B: Herbaceous plants-*Boehmeria penduliflora*.

The water-use parameters and water content values of the six tested plants are illustrated in Table 2 and Figure 4. The leaf IC, d, IWHC, WTR, and water content values of the tested plants were largely consistent, and the water contents of the herbaceous plants grown in the agricultural soil were significantly (*p* < 0.05) higher than those of the woody plants grown in the moderately rocky desertified soil. The results showed that the intracellular water metabolism and growth of herbaceous plants were more vigorous than those of woody plants, with lower IZ and IXc values, and higher IC, d, IWHC and WTR values. However, the leaf IWHTs of different plants were not significantly different. Moreover, the leaf IWUE of *Senecio scandens* was significantly (*p* < 0.05) higher than that of the other five plants, and there was also a significantly (*p* < 0.05) difference between the leaf IWUE of *Ipomoea batatas* and the IWUE of the remaining four plants.

### 3.3. Intracellular Water Utilization of Solanum Tuberosum and Capsicum Annuum

As shown in Table 3, the leaf IC, d, IWHC and WTR values of *S. tuberosum* were significantly (*p* < 0.05) higher than those of *C. annuum*, while *S. tuberosum* had lower (*p* < 0.05) IZ and IXc values. This result indicated that the good water conditions in the *S. tuberosum* leaves ensured a high WTR, growth rate, IC and d, and a low IZ and IXc values. Moreover, the water supply time (IWHT) of *C. annuum* leaves was increased by decreasing the WTR. However, the leaf IWUE of *S. tuberosum* was significantly (*p* < 0.05) lower than that of *C. annuum*. These results demonstrated that the leaf IWUE is closely related to the IWHT and that a long IWHT ensures the efficient utilization of intracellular water.

## 4. Discussion

Almost all life activities in plants are accompanied by charge separation, electron movement and proton and dielectric transport. In mesophyll cells, cells and organelles are both surrounded by the cell membrane, which is largely composed of lipids, proteins and sugars [1]. The phospholipid bilayer is the basic scaffold of the cell membrane, and can be divided into three layers: two electron density bands approximately 2.5 nm thick on the inside and outside of the membrane, and a transparent band approximately 2.5 nm thick in the middle [1,20]. Therefore, the cell can be regarded as a concentric sphere capacitor with both inductor and resistor functions, due to the outer and inner proteins in the cell membrane [19,22], in which the ions, ion groups and electric dipoles are equivalent to electrolytes of a capacitor. When a plant leaf is subjected to a clamping force stimulus (or environmental stress), the cell membrane permeability of the leaf cells changes instantly. Consequently, the concentration of electrolytes inside and outside the cell membrane also changes, which leads to variation in the leaf Z, Xc and C.

The Nernst equation is an equation used to quantitatively describe the electrical potential formed by ions between systems A and B, and can also theoretically be used to quantitatively describe the diffusion gradient of electrolytes inside and outside the cell membrane [21,22]. Using the Nernst equation, the Z (or Xc) = y$+ke^{-\mathrm{bF}}$ of the theoretical intrinsic relationships between the clamping force and leaf Z and Xc was revealed in this paper for the first time. The Gibbs free energy means that the reduced internal energy of the system can be converted into the work done by the system, and the Gibbs free energy of the leaf cell capacitor is theoretically equal to the work by clamping force. According to Gibbs free energy, the C=x+hF of the theoretical intrinsic relationships between the clamping force and leaf C was also revealed for the first time. The results demonstrated that the fitting equations of Z–F, Xc–F and C–F had good correlations, which highlighted the existence of the above-mentioned intrinsic mechanism.

In this study, the IZ, IXc, IC and d values of the intrinsic electrical indices in plant leaves were successfully detected for the first time using the intrinsic mechanical relationships between the clamping force and leaf Z, Xc and C. The results demonstrated that the leaf IC and d values of *B. papyrifera* grown in agricultural soil were significantly higher than those of *B. papyrifera* grown in moderately rocky desertified soil, and had lower IZ and IXc values. The leaf thickness in plants is highly variable as dependent mainly on plant species, the water state of leaf and maybe also on mineral nutrition [28,29]. The leaves of *B. papyrifera* grown under good water conditions had high IWHC and water contents, which supported the higher d or leaf thickness of *B. papyrifera* grown in agricultural soil. The herbaceous plants had higher IC and d, and lower IZ and IXc values than those of woody plants, and *S. tuberosum* leaves also had higher IC and d, and lower IZ and IXc values than those of *C. annuum.* These results well explain the phenomenon of life in plants—that is, when the intracellular water in plant leaves is sufficient and the concentration of electrolytes is low, plant cells have high IC and d, and low IZ and IXc.

Plants grown in karst areas often suffer from various degrees of karst ecological stresses (e.g., drought, high pH, and high bicarbonate contents) [30]. The results presented here showed that the leaves of *B. papyrifera* grown under good water conditions had high IWHC, WTR and water contents. The water supply time (IWHT) of the leaves under poor water conditions was increased by decreasing the WTR, and the IWUE was slightly different between leaves of the same plant species. This proposed plant adaptation mechanism under adverse environments is consistent with a previous study [31]. Herbaceous plants are more vulnerable to drought stress than woody plants, due to their shallow root distribution and inability to access deep water [32,33]. The results of this study showed that herbaceous plants grown in agricultural soil had higher leaf IWHC, WTR and water contents than the woody plants grown in moderately rocky desertified soil. These results also indicated that good water conditions for herbaceous plants supported their vigorous life activities, which is consistent with our general understanding of plant functions. However, the leaf IWHTs and IWUEs of the different plants were slightly different, which might have been due to the inherent characteristics of plants. For example, the significant leaf IWUE of *S. scandens* was likely related to its high leaf biomass. In this study, *S. tuberosum* had higher leaf IWHC and WTR and lower IWUE and IWHT than *C. annuum*, and these results are consistent with the biological fact that the biomass of *S. tuberosum* is higher than that of *C. annuum* [34,35].

Previous studies have shown that direct changes in plant electrical parameters such as Z and C can directly reflect changes in plant water [4,13,14,18,36,37]. However, the intracellular water movement, water-holding and utilization status of plant leaves cannot be obtained from the simple C, Z and Xc electrical parameters. In addition, the water status data from plant leaves obtained via photosynthesis-transpiration, turgor pressure, water potential, spectrometry, stable oxygen and hydrogen isotopes and other methods did not directly reflect the intracellular water status [24,25,26,27,38]. In this study, the IWHC, IWUE, IWHT and WTR of the intracellular water-use indices in plant leaves based on the plant’s intrinsic electrical parameters accurately revealed the life strategies and diversity of intracellular water metabolism in the leaves of the various experimental plants. Moreover, the indices for the method used in this study were obtained from the intrinsic electrical parameters in plant leaves, which overcame various drawbacks including the representativeness, stability and reproducibility inherent to the traditional impalement method. The intracellular water-use indexes IWHC, IWUE, IWHT and WTR based on plant intrinsic electrical parameters thus have promising potential as a novel method for routine monitoring of the intracellular water metabolism in plants.

## 5. Materials and Methods

### 5.1. Plant Materials

*Broussonetia papyrifera* plants were grown in two habitats, agricultural soil and moderately rocky desertified soil in Puding county, Guizhou Province (26°37′ N, 105°77′ E). *Amygdalus persica* L., *Rhus chinensis* Mill. and *Ginkgo biloba* L. were grown in moderately rocky desertified soil in Puding, and *Ipomoea batatas* (L.) Lam., *Senecio scandens* Buch.-Ham. ex D. Don and *Boehmeria penduliflora* Wedd. ex Long were grown in agricultural soil in Puding. *Capsicum annuum* L. and *Solanum tuberosum* L. were grown in potted agricultural soil at the Guizhou Vocational College of Agriculture in Qingzhen county, Guizhou Province (26°58′ N, 106°43′ E). The average annual temperature, sunshine hours and precipitation in Puding and Qingzhen counties were 15.1 and 14.1 °C, 1164.9 and 1128.2 h and 1378.2 and 1180.9 mm, respectively. The growth age, habitat information, measurement conditions and sampling weather of all tested plants are shown in Table 4. The vacuole volume basically represents the water-holding capacity of plant leaves, and the vacuoles of fully expanded leaf cells account for 50–90% of the cell volume. Thus, fully expanded leaves from fresh branches were measured as the experimental materials. Firstly, fully expanded leaves were taken from the third, fourth and fifth leaf positions of each branch, and the fresh leaves were immediately soaked in distilled water for 30 min. The water on the surface of the leaves was then removed. This soaking was to attain the full water saturation of the leaves which ensured that all tested leaves were comparable. Four branches of each plant were then measured. The tested leaves were sampled and measured at 8–10 a.m. on sunny days, and the measurement temperature was set to room temperature (25.0 ± 2.0 °C).

### 5.2. Lea f Z, Xc, and C Measurement

**Figure 5:**
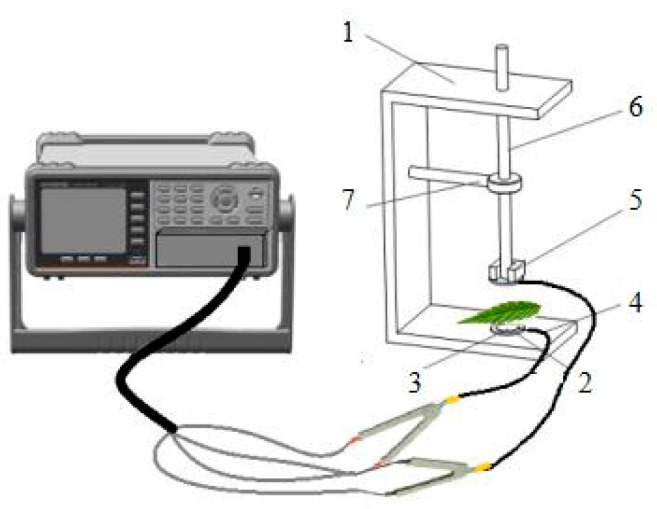
The experimental setup used in the study and a schematic diagram of the parallel-plate capacitor. 1: holder (315 mm in height), 2: cystosepiment (32 mm in diameter), 3: copper electrode plate (7 mm in diameter), 4: electrical conductor, 5: iron block, 6: plastic rod (295 mm in height), 7: bench holdfast (130 mm in length).

The leaf C and Z values were measured using a LCR tester (model 6300, Gwinstek, Taiwan, China), with a frequency and voltage of 3.0 kHz and 1.5 V, respectively, as used by Zhang et al. [16], with modifications. The center of the leaf was clipped between two copper electrodes in a homemade parallel-plate capacitor with a diameter of 7 mm (Figure 5). The leaf Z and C values were measured continuously at different clamping forces (1.139, 2.149, 3.178, 4.212, 5.245, 6.262 and 7.311 N), while adding iron blocks, and 11–13 data points were recorded at each clamping force. The 1 N of clipping force corresponded to the 0.26 bar of pressure on the plant leaves. The mesophyll cells are connected with plasmodesmata, thus, forming low-resistance routes for passing the electrical current. Most plant mesophyll cells can be divided into long cylindrical palisade tissue cells and irregularly spherical sponge tissue cells. In order to simplify the scientific problem, every mesophyll cell can be regarded as a concentric sphere capacitor. Thus, many aligned mesophyll cells (or symplast) comprise the leaf capacitor. To ensure that the voltage of each capacitor was consistent, the parallel connection mode of LCR was used. The leaf Xc was calculated according to Equation (29):

(29) $$\mathrm{Xc}=\frac{1}{2\pi \mathrm{fC}},$$

where π: 3.1416, f: frequency, and C: physiological capacitance.

Additionally, the leaf water contents were analyzed via the drying method, and the fresh weight of the leaves were measured before soaking.

### 5.3. Data Analyses

The data were analyzed using SPSS 18.0 (SPSS Inc., Chicago, IL, USA). A one-way analysis of variance followed by Duncan’s test was performed.

## 6. Conclusions

For the first time, the present work revealed the theoretical intrinsic relationships and fitting equations between clamping forces and leaf Z, Xc and C based on bioenergetics. The intrinsic electrical parameters (IZ, IXc and IC) of plant leaves were also detected for the first time using these equations. Subsequently, the IWHC, IWUE, IWHT and WTR of the intracellular water-use indices in the plant leaves were defined based on the plants’ intrinsic electrical parameters, and the metabolism physiology of intracellular water was evaluated in various plants. These indices accurately revealed the life strategies and diversity of intracellular water metabolism in plant leaves and could potentially be applied to the acquisition of plant intracellular water information. This study highlighted that the intrinsic electrical parameters of plants have promising potential as a novel technique for the rapid monitoring of plants’ physiological states.

## Figures and Tables

**Table 1:** Water-use parameters of *B. papyrifera* plants grown in the two soil conditions.

Habitats	IZ (MΩ)	IXc (MΩ)	IC (pF)	d	IWUE	IWHT	WTR
AS-B	0.50 ± 0.34 ^b^	1.13 ± 0.97 ^b^	99.48 ± 76.61 ^a^	67.60 ± 50.36 ^a^	0.12 ± 0.11 ^a^	27.22 ± 6.71 ^b^	40.80 ± 33.63 ^a^
MRDS-B	12.98 ± 10.75 ^a^	7.41 ± 0.83 ^a^	7.23 ± 0.70 ^b^	1.39 ± 0.77 ^b^	0.08 ± 0.06 ^a^	92.63 ± 84.12 ^a^	0.31 ± 0.13 ^b^

Note: Values indicate the mean ± SD, n = 12. Lower case letters indicate significant differences at a 5% level (*p* < 0.05). AS-B: *B. papyrifera* grown in agricultural soil, MRDS-B: *B. papyrifera* grown in moderately rocky desertified soil.

**Table 2:** Water-use parameters of the six plants.

Plants	IZ (MΩ)	IXc (MΩ)	IC (pF)	d	IWUE	IWHT	WTR
W-A	4.26 ± 1.53 ^b^	4.36 ± 1.50 ^b^	13.22 ± 3.65 ^d^	5.15 ± 1.43 ^e^	0.12 ± 0.06 ^c^	51.67 ± 1.10 ^a^	0.96 ± 0.40 ^d^
W-R	3.27 ± 0.93 ^c^	3.54 ± 0.82 ^c^	15.63 ± 3.18 ^c^	9.54 ± 2.30 ^c^	0.16 ± 0.06 ^bc^	48.49 ± 4.67 ^a^	1.33 ± 0.50 ^cd^
W-G	5.17 ± 1.14 ^a^	5.25 ± 1.08 ^a^	10.50 ± 2.11 ^e^	3.14 ± 0.94 ^f^	0.10 ± 0.04 ^c^	52.09 ± 1.62 ^a^	0.67 ± 0.21 ^d^
H-I	1.85 ± 0.44 ^e^	1.92 ± 0.50 ^e^	29.59 ± 8.62 ^a^	33.83 ± 14.89 ^a^	0.23 ± 0.13 ^b^	52.11 ± 9.86 ^a^	3.14 ± 1.07 ^a^
H-S	2.55 ± 0.89 ^d^	2.65 ± 1.00 ^d^	22.28 ± 7.12 ^b^	27.97 ± 8.12 ^b^	0.33 ± 0.21 ^a^	51.26 ± 1.71 ^a^	2.12 ± 1.00 ^bc^
H-B	2.82 ± 1.81 ^d^	3.31 ± 1.68 ^c^	19.68 ± 9.57 ^c^	6.00 ± 2.56 ^d^	0.09 ± 0.06 ^c^	43.79 ± 9.43 ^b^	2.38 ± 2.17 ^ab^

Note: Values indicate the mean ± SD, n = 12. Lower case letters indicate significant differences at a 5% level (*p* < 0.05). W-A: Woody plant-*Amygdalus persica*, W-R: Woody plant-*Rhus chinensis*, W-G: Woody plant-*Ginkgo biloba*, H-I: Herbaceous plants-*Ipomoea batatas*, H-S: Herbaceous plants-*Senecio scandens*, H-B: Herbaceous plants-*Boehmeria penduliflora*.

**Table 3:** Water-use parameters of the two plants grown in agricultural soil.

Plants	IZ (MΩ)	IXc (MΩ)	IC (pF)	d	IWHC	IWUE	IWHT	WTR
*S. tuberosum*	0.24 ± 0.10 ^b^	0.37 ± 0.21 ^b^	163.88 ± 49.86 ^a^	307.88 ± 94.13 ^a^	2164.13 ± 847.71 ^a^	0.19 ± 0.16 ^b^	34.99 ± 5.76 ^b^	62.23 ± 27.37 ^a^
*C. annuum*	4.03 ± 1.44 ^a^	4.12 ± 1.41 ^a^	14.26 ± 4.92 ^b^	72.29 ± 11.87 ^b^	55.84 ± 28.03 ^b^	1.69 ± 1.04 ^a^	51.70 ± 0.84 ^a^	1.09 ± 0.56 ^b^

Note: Values indicate the mean ± SD, n = 6. Lower case letters indicate significant differences at a 5% level (*p* < 0.05).

**Table 4:** Growth age, habitat information, measuring conditions and sampling weather for all tested plants.

Plants	Places	Age (Years)	Habitats	Soil Properties			Measurement Conditions		Sampling Weather
				pH	Organic Matter Content (g/kg)	Soil Moisture Content (%)	Time	Temperature (°C)	
*B. papyrifera* 1	Puding County	1	AS	6.27 ± 0.03	4.35 ± 0.65	18.46 ± 0.02	2018.08.25 a.m.	25.0 ± 2.0	Sunny
*B. papyrifera* 2		1	MRDS	6.85 ± 0.03	3.58 ± 0.33	15.51 ± 0.02			
*A. persica*		3	MRDS	6.74 ± 0.02	3.63 ± 0.15	15.66 ± 0.09	2018.08.24 a.m.		
*R. chinensis*		3	MRDS	6.81 ± 0.03	3.64 ± 0.27	16.13 ± 0.15			
*G. biloba*		3	MRDS	6.75 ± 0.04	3.57 ± 0.31	15.82 ± 0.12	2018.08.26 a.m.		
*I. batatas*		1	AS	6.31 ± 0.01	4.63 ± 0.21	18.53 ± 0.42			
*S. scandens*		1	AS	6.44 ± 0.02	4.98 ± 0.34	19.17 ± 0.21	2018.08.27 a.m.		
*B. penduliflora*		1	AS	6.47 ± 0.05	4.75 ± 0.36	19.21 ± 0.32			
*S. tuberosum*	Qingzhen County	1	PAS	6.32 ± 0.05	4.82 ± 0.53	19.65 ± 0.21	2018.08.15 a.m.		
*C. annuum*		1	PAS	6.34 ± 0.07	4.86 ± 0.31	19.72 ± 0.13			

Note: AS: agricultural soil, MRDS: moderately rocky desertified soil, PAS: potted agricultural soil.

## Supplementary Materials

The following are available online at https://www.mdpi.com/2223-7747/9/10/1256/s1. Additional File 1: Construction of the relationship models of clamping force (F) and leaf Xc, Additional File 2: The fitting equation parameters of *B. papyrifera* grown in two habitats, Additional File 3: The fitting equations of both the clamping force and the leaf Z, Xc, and C of *Brassica napus* in a phytotron, Additional File 4: Raw data.

## Abbreviations

C	capacitance
Z	impedance
Xc	capacitive reactance
F	clamping force
IC	intrinsic capacitance
IZ	intrinsic impedance
IXc	intrinsic capacitive reactance, d: specific effective thickness
IWHC	intracellular water-holding capacity,
IWUE	intracellular water-use efficiency
IWHT	intracellular water-holding time
WTR	water transfer rate
