# Supplementary material for: A Plant’s Electrical Parameters Indicate Its Physiological State: A Study of Intracellular Water Metabolism
Source: Plants (Basel). 2020 Sep 23;9(10):1256. doi: 10.3390/plants9101256 (PMC7598578; doi:10.3390/plants9101256)
Supplement: Supplementary file 1 [file plants-09-01256-s001.zip › supplementary-final/Additional file 1.pdf]

Additional file 1

Construction of the relationship model of clamping force (F) and leaf Xc.

Similar to Z, the concentration of electrolytes that respond to Xc inside and outside cell membrane determine the plant leaf Xc. The membrane permeability of electrolytes responding to Xc in plant cell membrane was different at different clamping forces. Similarly, the concentration differences in electrolytes responding to Xc inside and outside the cell membrane obey the Nernst equation and can be expressed as follows:

$$\mathrm{E - E^0 = \frac{R_0T}{n_{Xc}F_0} ln \frac{X_i}{X_o}} \qquad (1)$$

where E: the electromotive force (V), E^0: the standard electromotive force (V), R_0: the gas constant (8.314570 J K^{-1} mol^{-1}), T: the thermodynamic temperature (K), X_i: the concentration of electrolytes responding to Xc inside the cell membrane inside (mol L^{-1}), X_o: the concentration of electrolytes responding to Xc outside the cell membrane (mol L^{-1}), F_0: faraday constant (96485 C mol^{-1}), n_{Xc}: the number of transferred electrolytes (mol).

The internal energy of the electromotive force can be converted into pressure work, and they have a direct relationship, PV = a E:

$$\mathrm{PV = aE = a\,E^0 + \frac{a\,R_0T}{n_{XC}\,F_0} ln \frac{X_i}{X_o}} \qquad (2)$$

where P: the pressure intensity on the leaf cells (Pa), a: the energy conversion coefficient of the electromotive force, and V: the cell volume (m^3). $\mathrm{P = \frac{F}{S}}$, where F: the clamping force (N) and S: the effective area of the electrode plate (m^2).

For mesophyll cells, the sum of X_o and X_i is certain. X_i is directly proportional to the conductivity of electrolytes that respond to Xc, and the conductivity is the reciprocal of Xc. Hence, $\frac{X_i}{X_o}$ can be expressed as $\frac{X_i}{X_o} = \frac{\frac{L_0}{Xc}}{X - \frac{L_0}{Xc}} = \frac{L_0}{X\,Xc - L_0}$, where L_0: the ratio coefficient of the conversion between X_i and Xc, and X is $X_o + X_i$. Therefore, formula (2) was transformed into formula (3):

$$\mathrm{\frac{V}{S}F = a\,E^0 - \frac{a\,R_0T}{n_{XC}\,F_0} ln \frac{X\,Xc - L_0}{L_0}} \qquad (3)$$

Formula (3) was rewritten:

$$\mathrm{\frac{a\,R_0T}{n_{Xc}\,F_0} ln \frac{X\,Xc\text{-}L_0}{L_0} = a\,E^0 \text{-} \frac{V}{S}F} \qquad (4)$$

and

$$\ln\frac{X\,Xc\text{-}L_0}{L_0}=\frac{n_{Xc}F_0E^0}{RT}-\frac{V\,n_{Xc}F_0}{S\,a\,RT}F \qquad (5)$$

Formula (5) takes the exponents of both sides:

$$\frac{X\,Xc\text{-}L_0}{L_0}=e^{\frac{n_{Xc}\,F_0E^0}{R_0T}}\,e^{(-\frac{V\,n_{Xc}\,F_0}{Sa\,R_0T}F)} \qquad (6)$$

Further:

$$Xc=\frac{L_0}{X}+\frac{L_0}{X}e^{\frac{n_{Xc}\,F_0E^0}{R_0T}}\,e^{(-\frac{V\,n_{Xc}\,F_0}{S\,a\,R_0T}F)} \qquad (7)$$

Because $d=\frac{V}{S}$, formula (7) was transformed into::

$$Xc=\frac{J_0}{X}+\frac{J_0}{X}e^{\frac{n_{Xc}F_0E^0}{R_0T}}\,e^{(-\frac{d\,n_{Xc}F_0}{a\,R_0T}F)} \qquad (8)$$

For the same leaf tested in the same environment, the d, a, E^0, R_0, T, n_{Xc}, F_0, X, L_0 are constants. Because $p_0=\frac{L_0}{X}$, $k_2=\frac{L_0}{X}e^{\frac{n_{Xc}\,F_0E^0}{R_0T}}$, $b_2=\frac{d\,n_{Xc}\,F_0}{a\,R_0T}$, formula (8) was rewritten more simply:

$$Xc=p_0+k_2\,e^{-b_2F} \qquad (9)$$

where p_0, k_2 and b_2 are the model parameters.

When F=0, the intrinsic capacitive reactance (IXc) of the plant leaves can be obtained: $IXc=p_0+k_2$.
