# Supplementary material for: A Plant’s Electrical Parameters Indicate Its Physiological State: A Study of Intracellular Water Metabolism
Source: Plants (Basel). 2020 Sep 23;9(10):1256. doi: 10.3390/plants9101256 (PMC7598578; doi:10.3390/plants9101256)
Supplement: Supplementary file 1 [file plants-09-01256-s001.zip › supplementary-final/Additional file 2.pdf]

Additional file 2

The fitting equation parameters of *B. papyrifera* grown in two habitats

Habitats	Branch-leaf	Z-F			Xc-F			C-F		
		y_0/ k_1/ b_1	R^2	p<	p_0/ k_2/b_2	R^2	p<	x_0/h	R^2	p<
AS-B	1-3	0.05/0.33/0.28	0.9557	0.0001	0.05/0.65/0.26	0.9303	0.0001	49.11/39.66	0.9141	0.0001
	1-4	0.03/0.16/0.43	0.9811	0.0001	0.05/0.23/0.48	0.9789	0.0001	143.53/124.87	0.9857	0.0001
	1-5	0.03/0.10/0.36	0.9960	0.0001	0.05/0.21/0.45	0.9968	0.0001	195.54/95.64	0.9962	0.0001
	2-3	0.02/0.10/0.27	0.9370	0.0001	0.03/0.18/0.39	0.9616	0.0001	212.97/146.36	0.9293	0.0001
	2-4	0.04/0.21/1.35	0.9023	0.0001	0.05/0.38/1.45	0.9932	0.0001	562.84/94.05	0.9244	0.0001
	2-5	0.06/0.17/0.91	0.9791	0.0001	0.08/0.32/1.08	0.9942	0.0001	342.42/60.19	0.9403	0.0001
	3-3	0.11/0.86/0.84	0.9972	0.0001	0.20/2.39/1.13	0.9986	0.0001	61.37/33.49	0.9725	0.0001
	3-4	0.10/0.18/0.69	0.9918	0.0001	0.17/0.50/0.84	0.9904	0.0001	149.57/25.32	0.9774	0.0001
	3-5	0.18/0.88/1.05	0.9789	0.0001	0.24/1.24/1.09	0.9716	0.0001	103.27/19.95	0.9077	0.0001
	4-3	0.08/0.64/0.34	0.9878	0.0001	0.02/1.82/0.28	0.9845	0.0001	-7.66/26.93	0.9407	0.0001
	4-4	0.10/0.60/0.74	0.9908	0.0001	0.15/1.25/0.92	0.9872	0.0001	66.08/47.16	0.9643	0.0001
	4-5	0.13/0.80/0.22	0.9646	0.0001	0.05/3.24/0.21	0.9008	0.0001	10.55/7.41	0.9347	0.0001
MRDS-B	1-3	2.13/5.94/0.54	0.9082	0.0001	3.53/3.62/0.44	0.9912	0.0001	8.88/0.84	0.9473	0.0001
	1-4	1.43/5.27/0.54	0.9990	0.0001	2.29/4.18/0.38	0.9970	0.0001	9.29/1.68	0.9855	0.0001
	1-5	2.52/4.91/0.34	0.9970	0.0001	3.65/3.56/0.32	0.9968	0.0001	8.11/0.75	0.9700	0.0001
	2-3	1.17/6.94/0.22	0.9968	0.0001	2.95/4.83/0.20	0.9968	0.0001	6.86/0.85	0.9966	0.0001
	2-4	1.25/12.85/0.70	0.9859	0.0001	1.00/8.67/0.27	0.9730	0.0001	2.71/2.91	0.9283	0.0001
	2-5	1.11/7.83/0.45	0.9926	0.0001	2.34/5.61/0.35	0.9932	0.0001	7.02/1.75	0.9896	0.0001
	3-3	0.40/5.75/0.22	0.9853	0.0001	0.27/6.64/0.14	0.9849	0.0001	6.35/1.74	0.9374	0.0001
	3-4	4.36/30.58/0.37	0.9944	0.0001	3.10/3.94/0.13	0.9936	0.0001	7.57/0.53	0.9914	0.0001
	3-5	4.36/35.92/0.54	0.9974	0.0001	3.59/4.04/0.22	0.9986	0.0001	7.24/0.68	0.9916	0.0001
	4-3	1.05/5.94/0.20	0.9902	0.0001	2.98/3.96/0.20	0.9910	0.0001	7.82/0.80	0.9872	0.0001
	4-4	0.39/5.85/0.17	0.9736	0.0001	1.93/4.88/0.16	0.9884	0.0001	7.56/1.05	0.9833	0.0001
	4-5	1.46/7.07/0.37	0.9922	0.0001	2.49/4.84/0.29	0.9888	0.0001	7.44/1.40	0.9870	0.0001

Note: AS-B: Agricultural soil-*B. papyrifera*, MRDS: Moderately rocky desertification soil-*B. papyrifera*.
