# Supplementary material for: A Plant’s Electrical Parameters Indicate Its Physiological State: A Study of Intracellular Water Metabolism
Source: Plants (Basel). 2020 Sep 23;9(10):1256. doi: 10.3390/plants9101256 (PMC7598578; doi:10.3390/plants9101256)
Supplement: Supplementary file 1 [file plants-09-01256-s001.zip › supplementary-final/Additional file 3.pdf]

Additional file 3 The fitting equations of both the clamping force and the leaf Z, Xc, and C of *Brassica napus* in a phytotron

Objective: In order to verify the authenticity of the equations of both the clamping force (F) and the leaf Z, Xc, and C of the model organisms under the controlled experimental conditions.

Materials and methods: *Brassica napus* was grown in a phyotron (10× 5× 4 m) of State Key Laboratory of Environmental Geochemistry, Institute of Geochemistry, Chinese Academy of Sciences. Vermiculite mixed with perlite (2:1) was used as growth matrix of *Brassica napus*, and regularly supplement 1/2 times Hoagland nutrient solution. The culture temperature and illumination were respectively 25.0/19.0 ℃ (light/ dark) and 12h /12h (light/ dark), and illumination intensity was about 500μmol m^{-2} s^{-1}. *Brassica napus* plants with 6~7 fully expanded leaves were used for the determination. The leaf Z and C of the fully expanded leaves from the third leaf positions of each plant at different clamping forces were continuously measured, and 11-13 data points were recorded at each clamping force. The leaf Xc was calculated according to $Xc = \frac{1}{2\pi fC}$ (π= 3.1416, f= frequency, and C= physiological capacitance). The leaves were measured at 8~10 a.m., and the measurement temperature was room temperature (25.0±2.0 ℃).

Results: In our other study, the effects of *Sclerotinia sclerotiorum* infection on electrophysiology information of *Brassica napus*, and *Brassica napus* was grown under the controlled experimental conditions. As shown in Table AF3.1, the revealed fitting equations of both the clamping force (F) and the leaf Z, Xc, and C in this study were also better verified on *Brassica napus* in a phytotron, and their correlation coefficients (R^2) were all over 0.9930. This results here highlighted the authentic existence of the intrinsic mechanism relationships of F and leaf Z, Xc and C.

Table AF3.1 The fitting equation parameters of *Brassica napus* in the artificial weather room

Repeat-leaf position	Z-F			Xc-F			C-F		
	y_0/ k_1/ b_1	R^2	$p<$	p_0/ k_2/b_2	R^2	$p<$	x_0/h	R^2	$p<$
1-3	0.22/0.94/0.55	**0.9978**	0.0001	0.27/1.62/0.55	**0.9991**	0.0001	16.88/24.87	**0.9981**	0.0001
2-3	0.20/1.05/0.56	**0.9932**	0.0001	0.29/2.43/0.84	**0.9953**	0.0001	7.41/32.16	**0.9977**	0.0001
3-3	0.16/0.88/0.33	**0.9950**	0.0001	0.30/2.47/0.65	**0.9985**	0.0001	3.02/26.38	**0.9949**	0.0001

Table AF3.2 Raw data of the 1-3 leaf of *Brassica napus*

F (N)	C (pF)	Z(MΩ)	Xc (MΩ)	F (N)	C (pF)	Z(MΩ)	Xc (MΩ)
1.139	46.08	0.72	1.15	4.212	118.89	0.33	0.45
1.139	46.17	0.72	1.15	4.212	119.58	0.32	0.44
1.139	46.10	0.73	1.15	4.212	120.31	0.32	0.44
1.139	46.15	0.73	1.15	4.212	120.93	0.32	0.44
1.139	46.23	0.73	1.15	4.212	121.29	0.32	0.44
1.139	46.28	0.73	1.15	4.212	121.62	0.32	0.44
1.139	46.33	0.73	1.15	4.212	122.11	0.32	0.43
1.139	46.43	0.73	1.14	4.212	122.71	0.32	0.43
1.139	46.84	0.72	1.13	4.212	123.22	0.32	0.43
1.139	46.89	0.72	1.13	4.212	123.78	0.32	0.43
1.139	47.01	0.72	1.13	4.212	124.29	0.32	0.43
1.139	47.13	0.72	1.13	4.212	124.74	0.32	0.43
2.149	67.76	0.51	0.78	5.245	145.33	0.27	0.37
2.149	67.91	0.51	0.78	5.245	145.94	0.26	0.36
2.149	68.16	0.51	0.78	5.245	146.58	0.26	0.36
2.149	68.49	0.51	0.77	5.245	147.12	0.26	0.36
2.149	68.84	0.51	0.77	5.245	147.60	0.26	0.36
2.149	69.33	0.51	0.77	5.245	148.03	0.26	0.36
2.149	69.51	0.50	0.76	5.245	148.37	0.26	0.36
2.149	69.71	0.50	0.76	5.245	148.83	0.26	0.36
2.149	69.93	0.49	0.76	5.245	149.31	0.26	0.36
2.149	70.25	0.49	0.76	5.245	149.70	0.26	0.35
2.149	70.45	0.49	0.75	5.245	150.59	0.26	0.35
2.149	70.60	0.49	0.75				
3.178	93.23	0.38	0.57				
3.178	93.50	0.38	0.57				
3.178	93.79	0.38	0.57				
3.178	94.14	0.39	0.56				
3.178	94.44	0.39	0.56				
3.178	94.78	0.39	0.56				
3.178	95.05	0.39	0.56				
3.178	95.38	0.39	0.56				
3.178	95.63	0.39	0.55				
3.178	95.85	0.39	0.55				
3.178	96.11	0.39	0.55				
3.178	96.44	0.39	0.55				
3.178	96.69	0.39	0.55				

Table AF3.3 Raw data of the 2-3 leaf of *Brassica napus*

F (N)	C (pF)	Z(MΩ)	Xc (MΩ)	F (N)	C (pF)	Z(MΩ)	Xc (MΩ)
1.139	41.49	0.74	1.28	3.178	109.25	0.39	0.49
1.139	41.74	0.75	1.27	3.178	109.79	0.39	0.48
1.139	41.98	0.75	1.26	3.178	110.30	0.39	0.48
1.139	42.24	0.75	1.26	3.178	110.65	0.39	0.48
1.139	42.36	0.76	1.25	3.178	111.19	0.39	0.48
1.139	42.58	0.76	1.25	3.178	111.56	0.39	0.48
1.139	42.72	0.76	1.24				
1.139	42.80	0.77	1.24	4.212	138.35	0.31	0.38
1.139	43.03	0.77	1.23	4.212	139.02	0.31	0.38
1.139	43.22	0.77	1.23	4.212	139.56	0.31	0.38
1.139	43.50	0.77	1.22	4.212	139.94	0.31	0.38
1.139	43.83	0.77	1.21	4.212	140.26	0.31	0.38
1.139	44.01	0.77	1.21	4.212	140.88	0.31	0.38
1.139	44.21	0.77	1.20	4.212	141.40	0.31	0.38
1.139	44.46	0.77	1.19	4.212	141.97	0.31	0.37
1.139	44.67	0.77	1.19	4.212	142.47	0.31	0.37
				4.212	143.10	0.31	0.37
2.149	77.04	0.49	0.69	4.212	143.57	0.31	0.37
2.149	77.58	0.48	0.68	4.212	144.02	0.31	0.37
2.149	77.83	0.49	0.68	4.212	144.46	0.31	0.37
2.149	78.21	0.49	0.68	4.212	144.84	0.31	0.37
2.149	78.51	0.49	0.68				
2.149	78.67	0.49	0.67	5.245	172.59	0.24	0.31
2.149	78.83	0.49	0.67	5.245	173.40	0.24	0.31
2.149	79.01	0.50	0.67	5.245	174.30	0.24	0.30
2.149	79.18	0.50	0.67	5.245	174.93	0.24	0.30
2.149	79.33	0.50	0.67	5.245	175.47	0.24	0.30
2.149	79.47	0.51	0.67	5.245	176.06	0.24	0.30
2.149	79.72	0.51	0.67	5.245	176.63	0.24	0.30
2.149	79.95	0.51	0.66	5.245	177.31	0.24	0.30
2.149	80.13	0.51	0.66	5.245	177.90	0.24	0.30
2.149	80.35	0.51	0.66	5.245	178.40	0.24	0.30
				5.245	178.97	0.24	0.30
3.178	105.07	0.40	0.50	5.245	179.46	0.24	0.30
3.178	105.57	0.40	0.50	5.245	180.55	0.24	0.29
3.178	106.23	0.40	0.50				
3.178	106.84	0.40	0.50				
3.178	107.54	0.40	0.49				
3.178	108.10	0.40	0.49				
3.178	108.66	0.40	0.49				

Table AF3.4 Raw data of the 2-3 leaf of *Brassica napus*

F (N)	C (pF)	Z(MΩ)	Xc (MΩ)	F (N)	C (pF)	Z(MΩ)	Xc (MΩ)
1.139	35.47	0.68	1.50	4.212	108.41	0.35	0.49
1.139	35.49	0.69	1.49	4.212	108.76	0.35	0.49
1.139	35.51	0.70	1.49	4.212	109.76	0.35	0.48
1.139	35.79	0.70	1.48	4.212	110.48	0.34	0.48
1.139	35.69	0.71	1.49	4.212	111.11	0.34	0.48
1.139	35.73	0.72	1.48	4.212	111.56	0.34	0.48
1.139	35.73	0.72	1.48	4.212	112.38	0.34	0.47
1.139	35.63	0.73	1.49	4.212	112.83	0.34	0.47
1.139	35.65	0.74	1.49	4.212	112.82	0.34	0.47
1.139	35.80	0.74	1.48	4.212	112.75	0.34	0.47
1.139	36.04	0.74	1.47	4.212	113.05	0.34	0.47
2.149	57.34	0.54	0.93	5.245	141.71	0.27	0.37
2.149	57.57	0.54	0.92	5.245	142.77	0.27	0.37
2.149	58.02	0.54	0.91	5.245	143.84	0.27	0.37
2.149	58.22	0.53	0.91	5.245	144.38	0.27	0.37
2.149	58.41	0.54	0.91	5.245	144.68	0.27	0.37
2.149	58.43	0.54	0.91	5.245	145.09	0.27	0.37
2.149	58.42	0.55	0.91	5.245	145.29	0.26	0.37
2.149	58.62	0.55	0.90	5.245	145.81	0.26	0.36
2.149	59.05	0.55	0.90	5.245	146.20	0.26	0.36
2.149	59.28	0.55	0.90	5.245	146.46	0.26	0.36
2.149	59.66	0.55	0.89	5.245	146.85	0.26	0.36
2.149	59.98	0.55	0.88				
2.149	60.18	0.55	0.88				
2.149	60.30	0.55	0.88				
3.178	83.11	0.42	0.64				
3.178	83.57	0.42	0.63				
3.178	84.02	0.42	0.63				
3.178	84.37	0.42	0.63				
3.178	84.71	0.42	0.63				
3.178	85.41	0.42	0.62				
3.178	85.39	0.42	0.62				
3.178	85.83	0.41	0.62				
3.178	85.59	0.42	0.62				
3.178	85.62	0.42	0.62				
3.178	85.83	0.42	0.62				
3.178	86.03	0.42	0.62				
3.178	86.37	0.42	0.61				
